# Natural Naphthohydroquinone Dimer Rubioncolin C Exerts Anti-Tumor Activity by Inducing Apoptotic and Autophagic Cell Death and Inhibiting the NF-κB and Akt/mTOR/P70S6K Pathway in Human Cancer Cells

**DOI:** 10.3390/cells8121593

**Published:** 2019-12-07

**Authors:** Jia Wang, Ling Li, Jing Wang, Lihua Song, Ninghua Tan, Zhe Wang

**Affiliations:** Department of TCMs Pharmaceuticals, School of Traditional Chinese Pharmacy, China Pharmaceutical University, Nanjing 211198, China; 1731020094@stu.cpu.edu.cn (J.W.); 1821020435@stu.cpu.edu.cn (L.L.); 18851107621@163.com (J.W.); songlihua4835@163.com (L.S.)

**Keywords:** naphthohydroquinone dimer, rubioncolin C, anti-tumor activity, apoptosis, autophagy, NF-κB pathway, Akt/mTOR/P70S6K pathway

## Abstract

Naphthohydroquinone dimers isolated from *Rubia* plants have garnered more attention due to their distinctive chemical structures and intriguing bioactivities. In our previous studies, we obtained ten naphthohydroquinone dimers containing seven novel ones and found that most of them possessed anti-tumor activities, especially rubioncolin C. However, the underlying mechanism remains unknown. In this study, we focused on rubioncolin C and found that it could inhibit the growth of cancer cell lines with IC_50_ values between 1.14 and 9.93 μM. Further experiments demonstrated that rubioncolin C induced apoptotic and autophagic cell death and inhibited the Akt/mTOR/P70S6K signaling pathway in HCT116 and HepG2 cells. Moreover, we observed that rubioncolin C inhibited the TNF-α- and LPS-induced NF-κB activation upstream of the p65 protein, which contributed to rubioncolin C-induced cell death. Rubioncolin C could also prevent LPS-induced endotoxin shock in vivo. Moreover, rubioncolin C suppressed tumor growth through inducing apoptosis and autophagy and inactivating NF-κB in vivo. These findings clarify the anti-tumor mechanism of rubioncolin C using biochemical techniques and pharmacological models and might contribute to the future development of rubioncolin C as a new therapeutic agent for treating cancer.

## 1. Introduction

**Figure 1:**
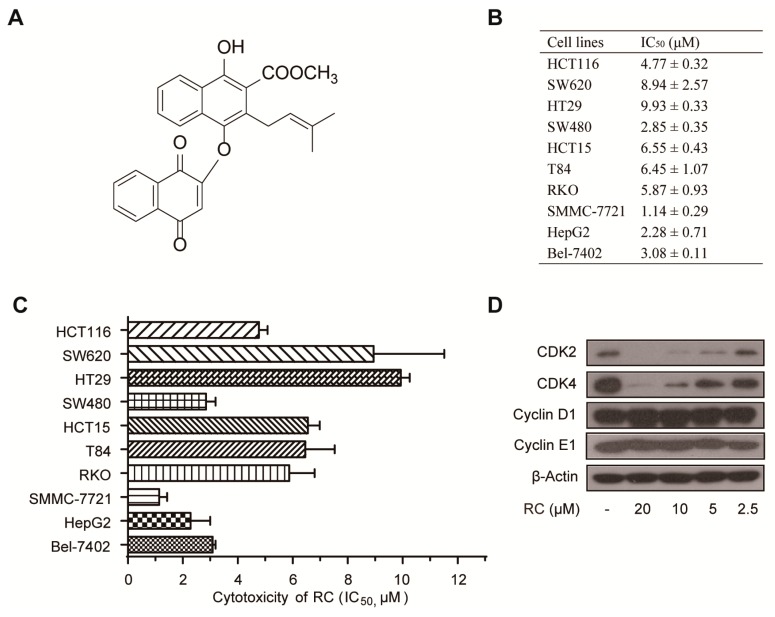
RC inhibits the growth of cancer cell lines. (**A**) The chemical structure and HPLC analysis of RC. (**B**,**C**) RC inhibited the growth of cancer cell lines and their IC_50_ values. HCT116, SW620, HT29, SW480, HCT15, T84, RKO, SMMC-7721, HepG2, or Bel-7402 cells were seeded in 96-well plates. After 24 h, the cells were incubated with various concentrations of RC for 48 h. The cell viability was determined by MTS assay. The data are presented as the means ± S.D. from three independent experiments. (**D**) RC influenced the expression of cell cycle regulating proteins. HepG2 cells were incubated with various concentrations of RC for 24 h. The cell lysates were prepared and subjected to a Western blot analysis with the indicated antibodies.

Plant-derived natural products with unique skeletons and various bioactivities, a rich source of privileged structures, drug leads and candidates, have played an irreplaceable role in drug discovery over the centuries, such as morphine, taxol, and artemisinin [1,2,3]. Quinones, including benzoquinones, naphthoquinones, phenanthraquinones and anthraquinones, widely exist in medicinal plants and exhibit various bioactivities, and some of their derivatives have been used in clinical therapy, such as mitoxantrone for anti-metastatic breast cancer, idebenone for anti-Alzheimer’s disease, and atovaquone for anti-pneumonia [4]. Among them, naphthohydroquinone dimers have drawn more attentions for their distinctive chemical structures and intriguing bioactivities [5]. The roots and rhizomes of *Rubia* plants, especially *R. cordifolia*, *R. yunnanensis* and *R. podantha*, have been widely used as traditional Chinese medicines for treating cancer, menoxenia, rheumatism and tuberculosis [6,7,8,9,10]. To date, 17 naphthohydroquinone dimers have been isolated from *Rubia* plants [5,11,12,13,14,15,16,17] (Figure S1), and some of them have been synthesized totally [18,19,20]. These isolated compounds possessed anti-tumor activities, particularly rubioncolin C (RC) (Figure 1A).

In general, the oncogenesis and development of cancer is associated with programmed cell death containing type I (apoptosis) and II (autophagy), both of which are genetically regulated and evolutionarily conserved processes that regulate cell fate [21]. Apoptosis, an important mechanism to induce cell death, has been considered as an effective strategy for cancer therapy. It involves the mitochondria-mediated intrinsic pathway and the death receptor-mediated extrinsic pathway, in which caspase-8 and -9 are the key initiative caspases, respectively [21]. Moreover, caspase-3 and PARP also play vital roles in cell apoptosis. Autophagy, an evolutionarily conserved catabolic process, is a lysosome-dependent pathway that involves the degradation of dysfunctional or redundant cytoplasmic constituents. It is also a key mechanism in various disease processes, especially tumorigenesis [21]. Increasing reports showed that autophagy acted as a double-edged sword against apoptosis, which could promote or suppress cancer cell death [22]. Therefore, it is worth exploring new compounds which induce apoptosis and influence autophagy.

The nuclear factor κB (NF-κB) signaling pathway is considered as a key regulator in many biological processes, such as cell proliferation, apoptosis, autophagy, and inflammation [23]. Mounting evidence has indicated that NF-κB is frequently abnormally activated in many diseases, such as cancer, diabetes and arthritis [24], which has led to the identification of more than 700 NF-κB inhibitors. But most of them have not been used in clinical therapy, except Bortezomib (Velcade), a reversible 26S proteasome inhibitor approved by the US FDA for treating multiple myeloma. In the last decade, we performed phytochemical investigations on nine *Rubia* plants and obtained ten naphthohydroquinone dimers containing seven novel ones [5,15,16]. Our previous studies also showed that naphthohydroquinone dimers possessed cytotoxic activities against ten tumor cell lines [5]. Among them, RC exhibited the best effects, but its underlying mechanisms remains unclear. In the current study, we firstly reported the novel finding that RC could inhibit tumor cell growth in vitro and in vivo and induce apoptotic and autophagic cell death through inhibiting the NF-κB and Akt/mTOR/P70S6K signaling pathways, which would contribute to the future development of RC as a new therapeutic agent for treating cancer.

## 2. Materials and Methods

### 2.1. Ethics Statement

Six- to eight-week-old female athymic nude BALB/c mice and BALB/c mice were purchased from Shanghai SLAC Laboratory Animal Co., Ltd. (Shanghai, China). The mice were maintained in specific pathogen-free (SPF) conditions at the China Pharmaceutical University. The animal experiments were conducted in strict accordance with the National Institutes of Health Guide for the Care and Use of Laboratory Animals. The protocol was approved by the Institutional Animal Care and Use Committee of China Pharmaceutical University and the Institutional Ethics Committee of China Pharmaceutical University (Approval Number: 2019-08-001; Approval date: May 2019).

### 2.2. Cell Lines and Culture

Human colorectal cancer cell lines (HCT116, SW620, HT29, SW480, HCT15, T84, RKO), and hepatocellular carcinoma cell lines (SMMC7721, HepG2, Bel7402), HEK293T, and RAW264.7 were purchased from the Type Culture Collection of Chinese Academy of Sciences (TCCCAS), Shanghai, China during the period from 2016 to 2018. All cell lines were authenticated by short tandem repeat profiling by TCCCAS before being purchased. HCT116, SW620, HT29, SW480, HCT15, and RKO cell lines were cultured in RPMI-1640 medium (Biological Industries, Kibbutz Beit-Haemek, Israel). SMMC7721, HepG2, Bel7402, HEK293T, and RAW264.7 cell lines were cultured in Dulbecco’s modified Eagle’s medium (DMEM, Biological Industries). T84 was cultured in DMEM/F12 (1:1) medium (Gibco). All media were supplemented with 10% (*v*/*v*) heat-inactivated fetal bovine serum (FBS, Biological Industries) and 1% (*v*/*v*) penicillin-streptomycin (Gibco) in a 37 °C incubator containing 5% CO_2_.

### 2.3. Chemicals, Reagents and Plasmids

Rubioncolin C (RC) was isolated from *Rubia podantha* by us, and the extraction and isolation procedures have been described previously [5]. Its purity and structure were confirmed by HPLC analysis (Figure S2) and ^1^H-, ^13^C-NMR and MS spectra (Figures S3–S5), respectively. RC was dissolved in dimethyl sulfoxide (DMSO) at 20 mM as a stock solution. Z-VAD-FMK was obtained from Selleckchem (USA). 3-MA, chloroquine, 5-fluorouracil, and LPS were purchased from Sigma (St. Louis, MO, USA). The reporter plasmids (5× κB-luciferase and pTK-Renilla) and the Flag-MyD88, Flag-TRAF2, HA-TRAF6, Flag-TAK1, HA-TAB1, HA-TAB2, Flag-IKKβ, HA-p65, GFP-LC3B plasmids have been described previously [25]. All of the plasmids were analytically verified by sequencing.

### 2.4. Cell Transfection and Luciferase Assay

HEK293T were seeded in 12- or 24-well plates and transiently transfected with indicated using Lipofectamine 2000 (Invitrogen). The luciferase reporter assays were performed as previously described [25].

### 2.5. MTS Assay

Cells were seeded at 5 × 10^3^ cells/well in 96-well plates containing 100 μL medium and cultured overnight followed by exposure to various concentrations of RC or DMSO for indicated times. Cell viability was determined by CellTiter 96^®^ AQueous One Solution Cell Proliferation Assay (MTS, Promega, Madison, WI, USA). Briefly, 20 μL of MTS was added to each well, and then the plate was incubated at 37 °C for 2 h in a humidified 5% CO_2_ atmosphere. Absorbance at 490 nm was measured using a 96-well plate reader (BioTek, Winooski, VT, USA). The results are presented as growth inhibition of 50% (IC_50_).

### 2.6. Western Blot Analysis and Antibody Information

The cells were lysed in lysis buffer (50 mM Tris, 150 mM NaCl, 1% Triton X-100 and 1 mM EDTA) supplemented with protease inhibitor (Roche, Indianapolis, IN, USA) and phosphatase inhibitor (Roche) in ice. Then the lysates were quantified using Detergent Compatible Bradford Protein Assay Kit (Beyotime, Hangzhou, China). Samples were equally subjected to SDS-PAGE and transferred to PVDF membranes (Millipore, Bedford, MA, USA). After blocking with 5% nonfat milk in TBST for 1 h, the membranes were incubated with the indicated primary antibodies at room temperature for 2 h or overnight at 4 °C. After washing three times with TBST, the membranes were then incubated with horseradish-peroxidase-conjugated secondary antibodies for 1 h at room temperature followed by membrane washing for another three times with TBST. The protein bands detection was carried out using the SuperSignal West Pico Chemiluminescent Substrate (Pierce, Waltham, MA, USA). The antibodies against PARP (9542, 1:2000), cleaved PARP (5625, 1:2000), Caspase-3 (14220, 1:2000), cleaved caspase-3 (9664, 1:2000), caspase-9 (9508, 1:2000), cleaved caspase-9 (52873, 1:2000), caspase 8 (9726, 1:2000), cleaved caspase-8 (9748, 1:2000), Bcl-2 (2870, 1:2000), LC3A/B (12741, 1:2000), LC3B (3868, 1:2000), IκBα (4812, 1:2000), phospho-IκBα (2859, 1:2000), phospho-NF-κB p65 (3033, 1:8000), phospho-mTOR (2971, 1:2000), phospho-Akt (4058, 1:2000), and phospho-P70S6K (9205, 1:2000) were purchased from Cell Signaling Technology (USA); the antibodies against p62/SQSTM (18420-1-AP, 1:2000), mTOR (20657-1-AP, 1:2000), P70S6K (14485-1-AP, 1:2000), Akt (10176-2-AP, 1:2000), and Lamin B1 (12987-1-AP, 1:2000), were purchased from Proteintech; the antibodies against HA (sc-7392, 1:2000) and p65 (sc-71675, 1:2000) were obtained from Santa Cruz.

### 2.7. Quantitative RT-PCR with Reverse Transcription

Total RNA was isolated from the RC untreated or treated cells or tissues using TRIzol reagent (Invitrogen) according to the manufacturer’s instructions and then was reverse transcribed using 2× Taq Master Mix (Vazyme, Nanjing, China). Quantitative real-time PCR for target genes was performed with SYBR^®^ Green Master Mix (High ROX Premixed) (Vazyme, Nanjing, China). The PCR primers used to detect the target genes are listed in Table S1.

### 2.8. Enzyme-linked Immunosorbent Assay (ELISA)

Murine cytokines IL-6 and TNF-α were detected by commercial ELISA kits (R&D Systems) following the manufacturer’s instructions.

### 2.9. Immunofluorescence Assay

Cells were fixed in 4% paraformaldehyde for 15 min followed by washing with PBS three times, and then permeated by 0.1% Triton X-100 for 20 min. After being washed with PBS three times, the cells were blocked with 5% BSA for 1 h and incubated with primary antibody p65 overnight at 4 °C. The cells were washed for another three times with PBS followed by incubation with a FITC-conjugated goat anti-mouse IgG (Jackson ImmunoResearch) for 1 h. The nuclei were counterstained with DAPI for 5 min at room temperature (Sigma). Images were captured by fluorescence microscope (Leica, Nussloch, Germany).

### 2.10. JC-1 Staining

Mitochondrial membrane potential of cells was measured using 5,5′,6,6′-tetrachloro-1,1′,3,3′-tetraethylbenzimidazolylcarbocyanine iodide (JC-1, Sigma) to detect mitochondrial depolarization occurring in the early stages of apoptosis. Cells were incubated with culture medium containing JC-1 (10 μg/mL) in a CO_2_ incubator at 37 °C for 30 min. The cells were washed for three times using PBS, and then were immediately monitored by fluorescence microscope (Leica).

### 2.11. Monodansylcadaverine and Lysotracker Red Staining

Monodansylcadaverine (MDC, Sigma) and Lysotracker red (Life Technologies, Carlsbad, CA, USA) were used to label autophagic vacuoles and lysosomes in cells, respectively. In brief, cells were incubated with 50 μM MDC or 100 nM Lysotracker red for 30 min at 37 °C. Then cells were washed with PBS three times and observed with a fluorescence microscope (Leica).

### 2.12. Annexin V-FITC/PI Double Staining Assay

Cell apoptosis was detected by flow cytometry using an Annexin V-FITC/PI staining kit (BD Biosciences, San Jose, CA, USA) according to the manufacturer’s instructions. Briefly, harvested cells were washed with cold PBS and resuspended in 500 μL Annexin V Binding Buffer. Then, the cells were incubated with 5 μL Annexin V-FITC and PI for 5 min at room temperature without light. Stained cells were analyzed by flow cytometry and data were analyzed using CELLQuest (Version 3.0, BD Biosciences, San Jose, CA, USA).

### 2.13. Preparation of RC-loaded Microemulsion

Briefly, 10 mg RC were completely dissolved in 1.2 mL caprylic capric triglyceride by ultrasound-assisted method for 1 h to obtain a clear solution. Then, 0.6 g polyethylene glycol and 1 g castor oil were mixed well by stirring for 30 min and the above clear solution was dropwise added in this mixed solution and stirred for 1 h. Then, the solution was passed through 0.22 μm filter membrane and the RC-loaded microemulsion was obtained.

### 2.14. Tumor Xenograft in Nude Mice and Toxicity Assessment

HepG2 cells with dimensions of 2 × 10^6^ HCT116 or 3 × 10^6^ were inoculated subcutaneously on the backs of female athymic nude BALB/c mice. After the tumor volumes reached 50 mm^3^, the tumor-bearing mice were randomly divided into several groups with five mice in each group. The mice were injected intraperitoneally with vehicle microemulsion or RC microemulsion every other day. Body weights were measured every other day throughout the treatment period. Tumor volumes were measured every other day and calculated using the following formula: tumor volume (mm^3^) = 0.52 × length × width^2^. The mice were killed at the end of the experiments. Tumor xenografts were removed and weighed. Part of each tumor tissue was stored in liquid nitrogen for quantitative RT-PCR analysis or at 4% formaldehyde solution for immunohistochemistry analysis. Heart, kidney, spleen, lung and liver from killed mice were also removed and fixed in the 4% formaldehyde solution for microscopic examination by hematoxylin-eosin staining. In addition, serum ALT, AST and creatine kinase were measured using corresponding kits (Nanjing Jiancheng, Nanjing, China) according to the manufacturer’s specific instructions.

### 2.15. Statistical Analysis

The Student’s *t*-test was used for the statistical analysis of the data. Mouse survival curve and statistics were analyzed with the log-rank (Mantel–Cox) test. All statistical analyses were performed using GraphPad Prism software. A *p* value < 0.05 was considered statistically significant.

## 3. Results

### 3.1. RC Inhibits the Growth of Cancer Cell Lines

The effect of RC on cell growth was performed using MTS assay on human colon carcinoma cell lines (HCT116, SW620, HT29, SW480, HCT15, T84, RKO) and human hepatocellular carcinoma cell lines (SMMC-7721, HepG2, Bel-7402). These cell lines were treated with various concentrations of RC for 48 h. The IC_50_ of RC, the concentration that induces 50% reduction of cell viability, was between 1.14 and 9.93 μM (Figure 1B,C). In the previous study, we found that RC exhibited the cell cycle arrest effect in HeLa cells by flow cytometry [5], which drove us to determine whether RC could influence the expression of cell cycle regulating proteins. As shown in Figure 1D, RC dose-dependently inhibited the expression of CDK2 and CDK4. These results demonstrate that RC inhibits the growth of cancer cell lines.

### 3.2. RC Induces Apoptosis in HCT116 and HepG2 cells

**Figure 2:**
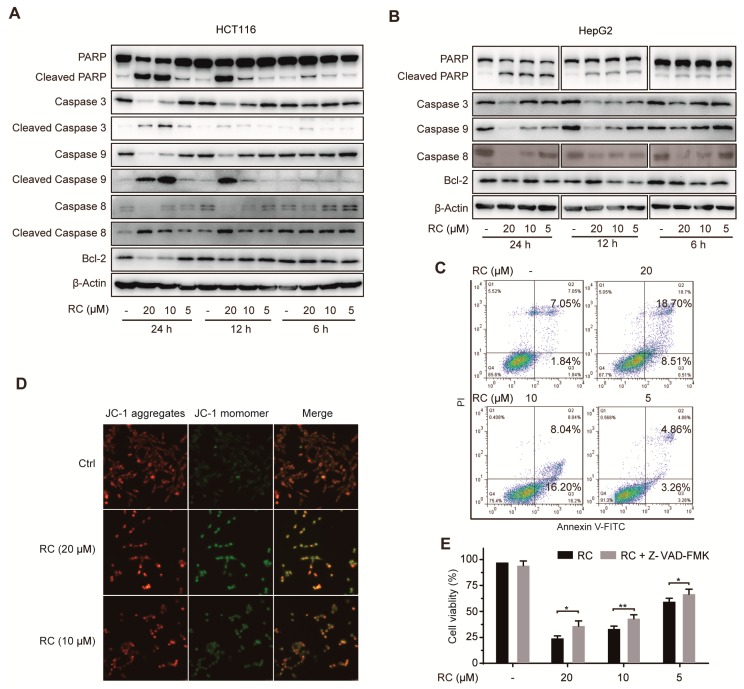
RC induces apoptosis in HCT116 and HepG2 cells. (**A**,**B**) RC influenced the expression of apoptosis-related proteins. HCT116 or HepG2 cells were incubated with various concentrations of RC for 24, 12, or 6 h and the expression of apoptosis-related proteins was measured by Western blotting. (**C**,**D**) HepG2 cells were incubated with various concentrations of RC for 24 h and the cells were co-stained with Annexin V/PI to determine apoptosis by flow cytometry (**C**), or stained with JC-1 and photographed by fluorescence (**D**). (**E**) HepG2 cells were incubated with or without Z-VAD-FMK (25 μM) for 1 h, and then treated with various concentrations of RC for 24 h. Cell viability was determined by MTS assay. The data are presented as the means ± S.D. from three independent experiments. *, *p* < 0.05; **, *p* < 0.01.

**Figure 3:**
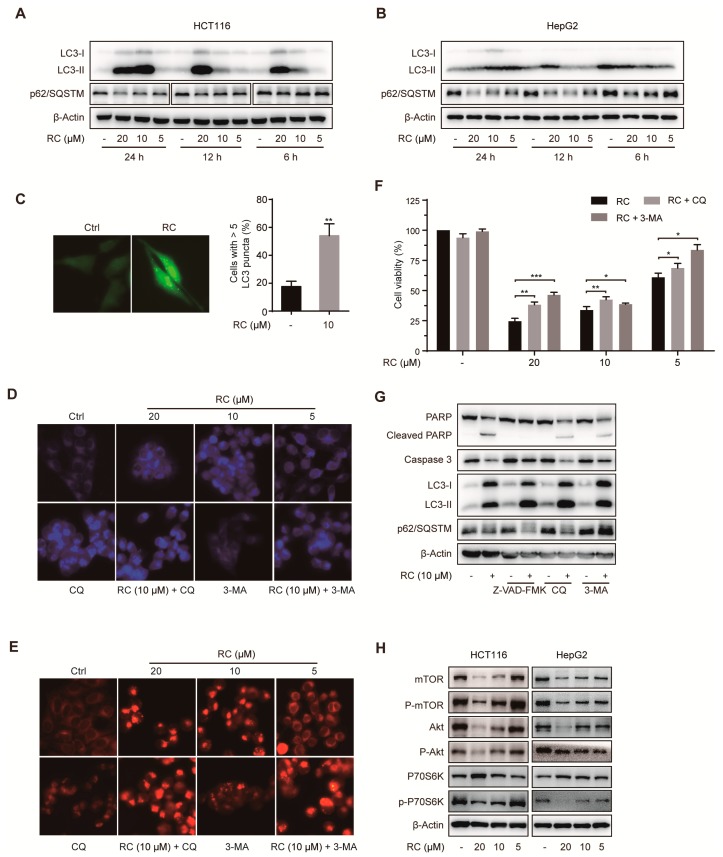
RC induces autophagic cell death by inhibiting the Akt/mTOR/P70S6K signaling pathway in HCT116 and HepG2 cells. (**A**,**B**) RC influenced the expression of autophagy-related proteins. HCT116 or HepG2 cells were treated with various concentrations of RC for 24, 12, or 6 h, and the expression of autophagy-related proteins was determined by Western blotting. (**C**) RC increased the aggregation of GFP-LC3 dots. HepG2 cells were transfected with GFP-LC3 plasmid. Twenty-four hours after transfection, the cells were incubated with 10 μM RC for 12 h, and the GFP-LC3B distribution was analyzed and quantified with a fluorescence microscope. Only cells with more than five puncta were counted. (**D**) RC increased the amounts of autophagic vesicles. HepG2 cells were incubated with or without 3-MA (3 mM) or CQ (25 μM) for 1 h, and then treated with various concentrations of RC for 12 h. After being stained with MDC for 30 min at 37 ^o^C, cells were imaged with a florescence microscope. (**E**) RC increased the amounts of lysosome or induced lysosome aggregation. Similarly to (**D**), cells were stained with Lysotracker red for 30 min at 37 ^o^C and captured by florescence microscope. (**F**–**G**) RC induced autophagic cell death. HCT116 were incubated with or without 3-MA (3 mM) or CQ (25 μM) for 1 h, and then treated with various concentrations of RC for 24 h. The cell viability was evaluated by MTS assay (**F**), or the expression of related proteins was determined by Western blotting (**G**). (**H**) RC decreased the expression of mTOR, phospho-mTOR, Akt, phospho-Akt, and phospho-P70S6K. HCT116 or HepG2 cells were treated with various concentrations of RC for 24 h; the cell lysates were prepared and Western blotting with the indicated antibodies. The data in (**C**) and (**F**) are presented as the means ± S.D. from three independent experiments. *, *p* < 0.05; **, *p* < 0.01, ***, *p* < 0.001.

To investigate the potential mechanism of RC to inhibit the growth of human colon carcinoma cell lines and human hepatocellular carcinoma cell lines, we chose HCT116 and HepG2 cell lines for the further study, respectively, based on the IC_50_ of RC and their universalities in the preclinical study. Firstly, we evaluated the effects of RC on several apoptosis-related proteins by Western blotting and found that RC dose- and time-dependence inhibited the expression of PARP, caspase -3, -8, -9, and Bcl-2 (Figure 2A,B). Consistently, RC could promote the cleavage of PARP, caspase -3, -8, and -9 (Figure 2A,B). We also found that RC increased both the Annexin V-FITC and Annexin VFITC/PI positive cells (Figure 2C), which demonstrates that it resulted in both early and late apoptosis. Furthermore, by the fluorometric analysis of JC-1 stained cells, we observed that RC disrupted the mitochondrial membrane potential (Figure 2D). Moreover, Z-VAD-FMK, a specific apoptosis inhibitor targeting the pan-caspases, not only blocked RC-induced cell death (Figure 2E), but also inhibited RC-induced the cleavage of PARP and the decrease of caspase 3 (Figure 3G), which suggests that caspase activation was required in this process. Collectively, these results demonstrate that RC induces apoptosis in HCT116 and HepG2 cells.

### 3.3. RC Induces Autophagic Cell Death Through Inhibiting Akt/mTOR/P70S6K Signaling Pathway in HCT116 and HepG2 cells

Autophagy is an important catabolic pathway for damaged organelles, lipids and misfolded proteins in cells, and is involved in cell survival and drug resistance. Therefore, we investigated whether RC influenced autophagy by different methods. The lipidation of microtubule-associated protein LC3-I, an autophagy marker, is converted to LC3-II during autophagy. RC significantly increased the expression of LC3-II (Figure 3A,B), which could be further enhanced by chloroquine (CQ) (Figure 3G), a late-stage autophagy inhibitor. Consistently, RC also increased the aggregation of GFP-LC3 dots (Figure 3C). Sequestosome 1 (SQSTM1, p62), a ubiquitin binding protein, is reduced through lysosomal degradation of autophagosomes during autophagy. As shown in Figure 3A,B, RC decreased the expression of p62/SQSTM1 in a dose-dependent manner. In addition, monodansylcadaverine (MDC) staining showed that RC led to an obvious increase of autophagic vesicles with or without treatment with CQ or 3-methyladenine (3-MA, an early-stage autophagy inhibitor) (Figure 3D). Similar results were also observed by lysosomal staining (Figure 3E), which demonstrated that RC increased the amount of lysosome or induced lysosome aggregation. These results support that RC causes autophagy in HCT116 and HepG2 cells.

Because RC-induced cell death was only modestly rescued by Z-VAD-FMK, the link between the autophagy and cell viability was investigated. We found that autophagy inhibitors, especially 3-MA, obviously rescued cell death induced by RC (Figure 3F) and inhibited the cleavage of PARP and caspase 3 (Figure 3G), which demonstrates that RC induces autophagic cell death. Given the critical role of the Akt/mTOR/P70S6K signaling pathway in autophagy, we investigated the effect of RC on the expression of this pathway associated proteins. As shown in Figure 3H, mTOR, phospho-mTOR, Akt, phospho-Akt, and phospho-P70S6K were dose-dependently decreased. Collectively, these results reveal that RC induces autophagic cell death through inhibiting the Akt/mTOR/P70S6K signaling pathway in HCT116 and HepG2 cells.

### 3.4. RC Inhibits the Activation of NF-κB Signaling Pathway In Vitro and In Vivo

**Figure 4:**
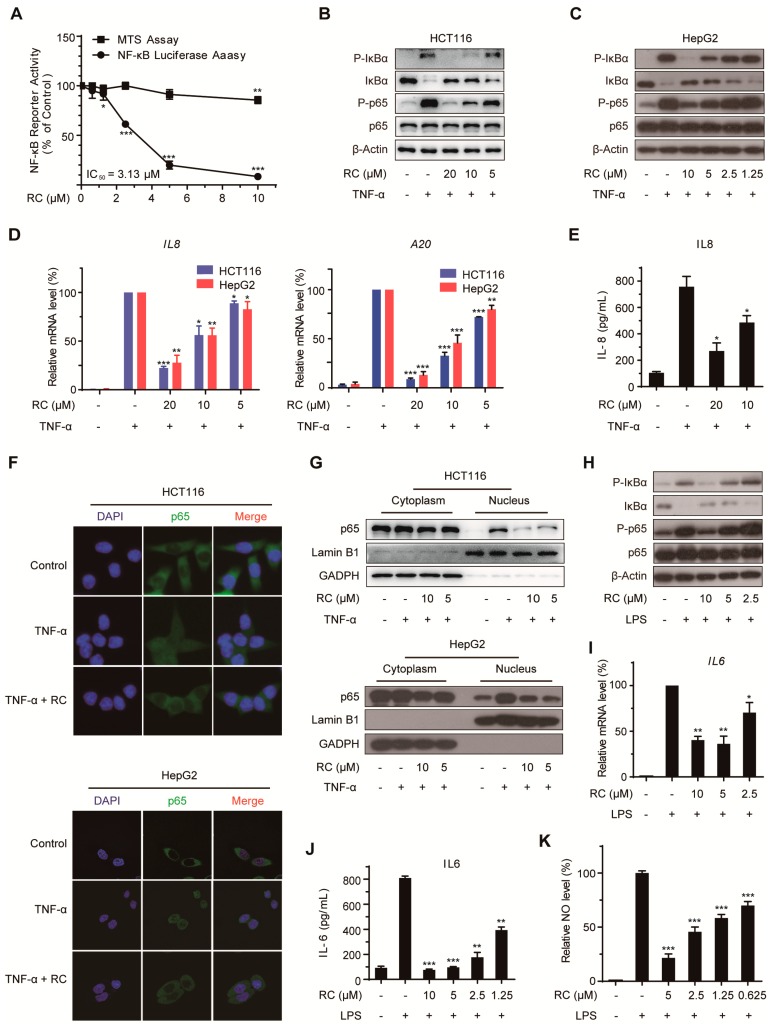
RC inhibits the activation of NF-κB signaling pathway. (**A**) RC inhibited the NF-κB signaling pathway. HEK293T cells were transfected with the 5× κB-luciferase and pTK-Renilla reporters. Twenty-four hours after transfection, the cells were treated with various concentrations of RC for 6 h, and then incubated with 10 ng/mL TNF-α for 4 h before the luciferase activity assay and MTS assay. (**B**,**C**) RC reduced TNF-α-induced IκBα phosphorylation, IκBα degradation and p65 phosphorylation. HCT116 or HepG2 cells were incubated with various concentrations of RC for 24 h and treated with 10 ng/mL TNF-α for 10 min. The cell lysates were prepared and subjected to a Western blotting analysis with the indicated antibodies. (**D**) RC inhibited TNF-α-induced expression of NF-κB target genes. HCT116 or HepG2 cells were treated with various concentrations of RC for 24 h and stimulated with 10 ng/mL TNF-α for 2 h. The expression of the NF-κB target genes, *IL-8* and *A20* were measured by quantitative RT-PCR and normalized to GADPH expression. (**E**) RC inhibited TNF-α-induced IL-8 production. HepG2 cells were treated with RC for 24 h before treatment with 10 ng/mL TNF-α for 4 h. The culture supernatant was collected and subjected to ELISA analysis. (**F**,**G**) RC inhibited the TNF-α-induced nuclear translocation of p65. HCT116 or HepG2 cells were incubated with 10 μM RC for 6 h and treated with 10 ng/mL TNF-α for 15 min, and then subjected to an immunocytochemical analysis or a nuclear and cytoplasmic protein extraction analysis. (**H**,**I**) RC inhibited LPS-induced IκBα phosphorylation, IκBα degradation, p65 phosphorylation, and *IL-6* mRNA expression. RAW264.7 cells were treated with various concentrations of RC for 24 h and treated with 1 μg/mL LPS for 3 h. The proteins and *IL-6* mRNA expression were determined. (**J**,**K**) RC inhibited LPS-induced IL-6 and NO production. RAW264.7 cells were treated with various concentrations of RC for 24 h and treated with 1 μg/mL LPS for 24 h. The production of IL-6 and NO was measured. The data in (**D**), (**E**), and (**I**–**K**) are presented as the means ± S.D. from three independent experiments. *, *p* < 0.05; **, *p* < 0.01; ***, *p* < 0.001.

NF-κB signaling pathway plays a critical role in many cancers and is involved in many biological processes, such as cell proliferation, apoptosis and autophagy. Using NF-κB-dependent luciferase assay in HEK293T cells, RC dose-dependently inhibited TNF-α-induced NF-κB activation with a IC_50_ value of 3.13 μM and did not exhibit obvious cytotoxicity (Figure 4A), which prompted us to explore the effect of RC on this pathway in HCT116 and HepG2 cells. The effect of RC on the expression of NF-κB-associated proteins was examined using Western blotting and the results show that RC reduced TNF-α-induced IκBα phosphorylation, IκBα degradation and p65 phosphorylation (Figure 4B,C). Furthermore, we found that the TNF-α-induced expression of *IL-8* and *A20* mRNAs was also decreased by RC (Figure 4D). Consistently, RC dose-dependently suppressed the TNF-α-induced production of IL-8 (Figure 4E). In addition, the effect of RC on the p65 nuclear translocation induced by TNF-α was investigated by immunofluorescence assay and a nuclear and cytoplasmic protein extraction analysis. As shown in Figure 4F,G, RC interrupted TNF-α-induced p65 nuclear translocation. As expected, similar results were also observed in LPS-induced NF-κB activation in RAW264.7 cells. The changes of phospho-IκBα, IκBα, phospho-p65, *IL6* mRNA, IL-6, and NO induced by LPS were blocked (Figure 4H–K). Taken together, these results demonstrate that RC inhibits TNF-α- and LPS-induced activation of NF-κB signaling pathway in vitro. 

**Figure 5:**
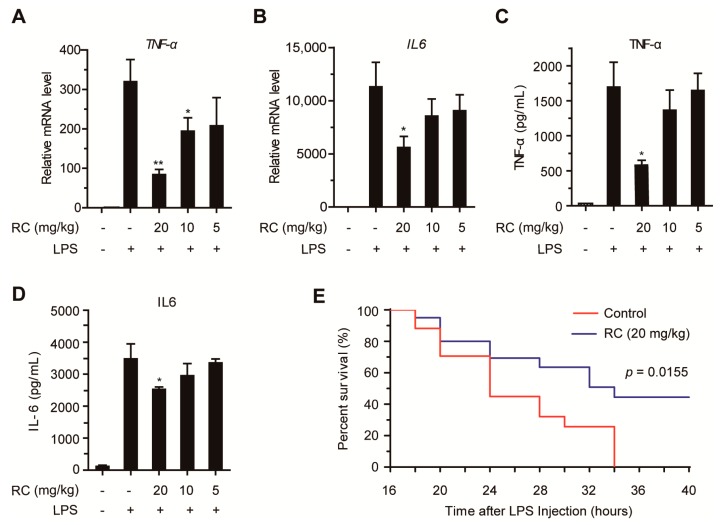
RC prevents endotoxic shock in BALB/c mice. (**A**–**D**) RC prevents endotoxic shock in vivo. Mice (*n* = 6 per group) were intraperitoneally injected with control microemulsion or RC microemulsion for 4 h before intraperitoneal injection of LPS (10 mg/kg). One hour later, the relative levels of *TNF-α* and *IL-6* mRNAs in the liver were evaluated by quantitative RT-PCR (**A**,**B**), the serum TNF-α and IL-6 levels were determined by ELISA (**C**,**D**). (**E**) RC improved animal survival. Mice (*n* = 15 per group) were intravenously injected with control microemulsion or RC microemulsion (20 mg/kg) for 4 h before intraperitoneal injection of LPS (20 mg/kg). Animal survival was recorded in two or four h intervals (*p* = 0.0155, Mantel-Cox test). The data are presented as the means ± S.D. *, *p* < 0.05; **, *p* < 0.01.

To investigate the effect of RC on the NF-κB signaling pathway in vivo, the mouse endotoxin shock model induced by LPS was established in BALB/c mice. The mice were intraperitoneally injected with PBS or LPS (10 mg/kg) after they were intraperitoneally injected with control microemulsion or RC microemulsion for 4 h. One hour after LPS injection, the production of pro-inflammatory cytokines was evaluated. As shown in Figure 5A,B, RC inhibited the expression of TNF-α and IL6 mRNAs in liver tissue of RC-treated mice. Consistently, the expression of TNF-α and IL6 in the serum was also decreased in RC-treated mice (Figure 5C,D). Moreover, the survival experiment of mice injected with a lethal dose of LPS (20 mg/kg) was performed, and the results showed that nearly 40% of the RC-treated mice survived whereas all of the control mice died within 34 h (Figure 5E). Collectively, these results demonstrate that RC inhibits the activation of NF-κB signaling pathway in vivo.

### 3.5. RC Represses NF-κB Activation Upstream of the p65 Protein and Contributes to Cell Death

**Figure 6:**
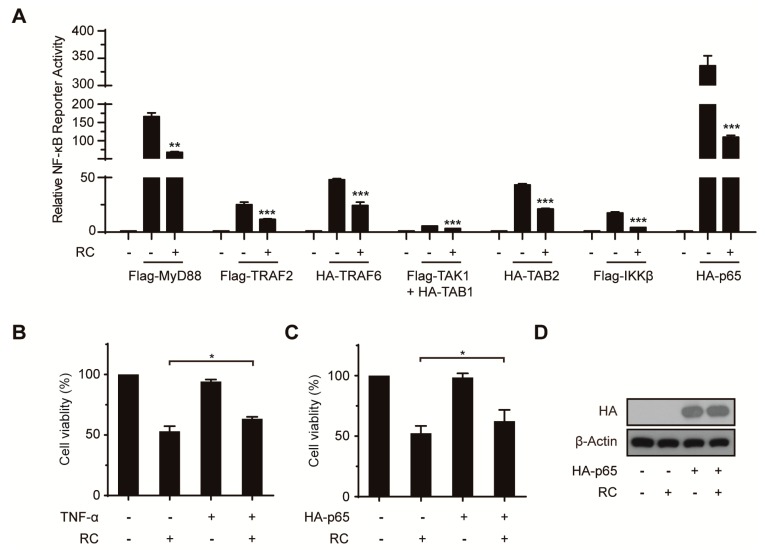
RC represses NF-κB activation upstream of the p65 protein and contributes to cell death. (**A**) RC inhibits NF-κB activation caused by overexpressing NF-κB-associated key proteins. The indicated plasmids were transfected into HEK293T cells together with the 5× κB-luciferase and pTK-Renilla reporters. Twenty-four hours after transfection, the cells were incubated with 10 μM RC for 6 h before luciferase assays were performed. (**B**–**D**) RC induced cell death involving in NF-κB pathway. HepG2 cells were incubated in the presence or absence of TNF-α (20 ng/mL, 30 min) prior to treatment with 5 μM RC for 24 h. The cell viability was evaluated by MTS assay (**B**). Similarly to (**B**), cells were transfected with HA-p65 plasmid for 24 h, then incubated with 5 μM RC for 24 h. The cell viability was also evaluated by MTS assay (**C**,**D**). *, *p* < 0.05; **, *p* < 0.01; ***, *p* < 0.001.

Several key proteins including MyD88, TRAF2, TRAF6, TAK1, TAB1, TAB2, IKKβ and p65 were involving in the canonical NF-κB activation induced by TNF-α or LPS. To investigate where RC acts in this pathway, Flag-MyD88, Flag-TRAF2, HA-TRAF6, Flag-TAK1, HA-TAB1, HA-TAB2, Flag-IKKβ, or HA-p65 were transiently transfected into HEK293T cells together with the 5× κB-lucieferase and pTK-Renilla reporters. RC inhibited the NF-κB reporter activity in cells transfected with any above-mentioned plasmid (Figure 6A). Combined with the Figure 4F, these results suggest that RC inhibits the activation of the NF-κB signaling pathway upstream of the p65 protein. In view of the good positive correlation between the cytotoxicity and NF-κB inhibited activity of RC in vitro, the NF-κB pathway was activated by TNF-α or p65 plasmid to explore its role in RC-induced cell death. As shown in Figure 6B–D, cells pretreated with TNF-α or overexpressing p65 resulted in the inhibition of RC-induced cell death compared with RC alone, which indicates that RC induces cell death involving in the inactivation of the NF-κB pathway.

### 3.6. RC Inhibits Tumor Growth and Induces Apoptosis and Autophagy with Inhibition of NF-κB In Vivo

**Figure 7:**
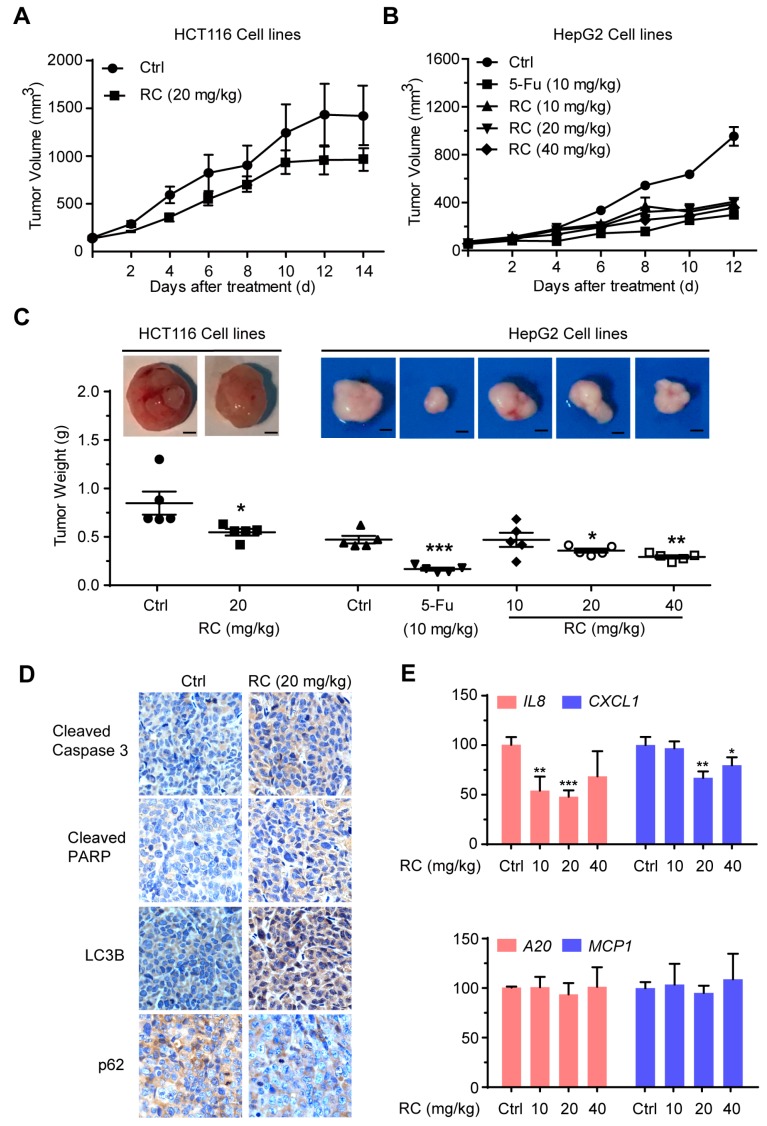
RC inhibits tumor growth and induces apoptosis and autophagy with the inhibition of NF-κB in vivo. (**A**–**C**) RC inhibited the growth of HCT116 and HepG2 xenograft tumors. Female athymic nude BALB/c mice bearing HCT116 (*n* = 5) or HepG2 (*n* = 5) xenograft tumors were intraperitoneally injected with control microemulsion or RC microemulsion every other day. 5-FU (10 mg/kg) group as positive control. Effects of RC on the growth curves of subcutaneous xenografts (**A**,**B**) and on the tumor weight (**C**) in the HCT116 and HepG2 models. (**D**) The expression of the apoptosis and autophagy related proteins in HepG2 tumor tissues was evaluated by an immunohistochemistry analysis. (**E**) The expression of the NF-κB target genes, *IL-8*, *CXCL-1*, *A20* and *MCP-1* in HepG2 tumor tissues was determined by quantitative RT-PCR and normalized to *GADPH* expression. *, *p* < 0.05; **, *p* < 0.01; ***, *p* < 0.001.

**Figure 8:**
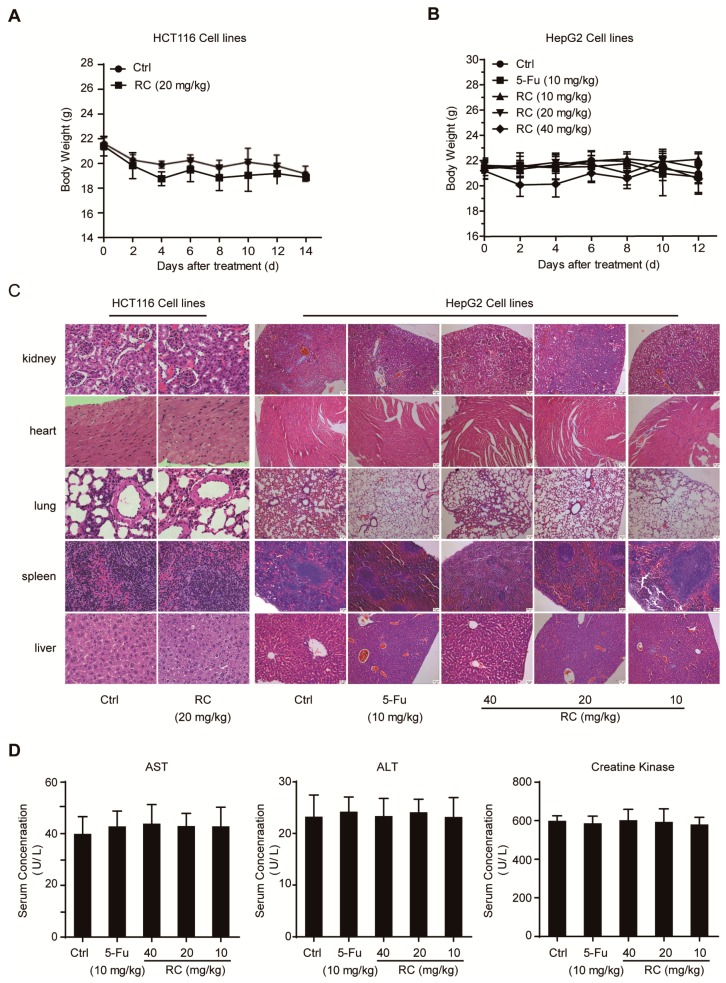
Analysis of potential side effects for treated with RC. (**A**,**B**) The change curves of body weights of BALB/c bearing HCT116 (*n* = 5) (**A**) or HepG2 (*n* = 5) (**B**) xenograft tumors. (**C**) Representative hematoxylin-eosin staining of heart, kidney, spleen, lung and liver from vehicle- and RC-treated groups. (**D**) The evaluation of serum ALT, AST and creatine kinase for vehicle- and RC-treated groups.

The anti-tumor activities of RC in vivo were investigated in HCT116 and HepG2 tumor models. Compared with the control, administration of RC microemulsion in mice via intraperitoneal injection every other day resulted in the inhibition of tumor growth in these two models by measuring tumor volume and weight (Figure 7A–C). The possible toxicity of RC was also evaluated by several manners. As shown in Figure 8A–D, mice in the RC-treated groups maintained normal weights and did not show any abnormality in main organs and the levels of serum ALT, AST and creatine kinase. Furthermore, the immunohistochemistry analysis for apoptosis- and autophagy-associated marker proteins in the tumor tissues shows that RC promoted the expression of cleaved caspase-3, cleaved PARP and LC3B, while it reduced the p62 content (Figure 7D). In addition, the quantitative RT-PCR analysis exhibited that the expression of NF-κB target genes, *IL-8* and *CXCL-1*, was reduced in RC-treated groups compared with the control (Figure 7E). Taken together, these results demonstrate that RC inhibits tumor growth and induces apoptosis and autophagy with inhibition of NF-κB in vivo.

## 4. Discussion

Natural products, possessing enormous structural and chemical diversity, inspire novel discoveries in chemistry, biology, and medicine, such as penicillin, avermectin and artemisinin [26]. The roots and rhizomes of several *Rubia* plants have been widely used for the treatment of cancer, menoxenia, rheumatism, and tuberculosis, especially *R. cordifolia*, which was recorded as a traditional Chinese medicine in all editions of Chinese Pharmacopeia. In previous studies, we have obtained nearly 200 compounds from nine *Rubia* plants, including *R. cordifolia*, *R. yunnanesis*, *R. podantha*, *R. schumanniana*, *R. alata*, *R. wallichiana*, *R. oncotricha*, *R. sylvatica*, and *R. ovatifolia*, and investigated their bioactivities and mechanisms of action, including Rubiaceae-type cyclopeptides [6,7,8,9,10], quinones [5,15,16,27,28,29], triterpenes [27,30,31]. For their unique structures and preferable bioactivities, naphthohydroquinone dimers have attracted our attention, especially rubioncolin C (RC) which exhibits the best cytotoxicity among them. In this study, we focused on RC and clarified the underlying anti-tumor mechanisms for the first time using biochemical techniques and pharmacological models. 

Firstly, we evaluated the cytotoxicity of RC on human colon carcinoma cell lines and human hepatocellular carcinoma cell lines using MTS assay and found that RC is a potent inhibitor of cell growth with IC_50_ values between 1.14 and 9.93 μM. In view of the important role of cell cycle arrest in tumor cell growth inhibition, the expression of several cell cycle-related proteins was investigated and RC influences the expression of CDK2 and CDK4, which suggests the feasibility of cell cycle arrest in G_0_/G_1_ and G_2_/M. Both apoptosis and autophagy are important mechanisms of cell death, which prompted us to explore the effects of RC on these two biological processes. Several biochemical assays, including Western blot, fluorescence detection, flow cytometry and cell viability showed that RC induced apoptosis in HCT116 and HepG2 cells, which could be blocked partially by caspases inhibitor Z-VAD-FMK. RC also triggered autophagy and inhibited the Akt/mTOR/P70S6K signaling pathway which play a critical role in autophagy. Considering the double-edged sword role of autophagy in tumor cell survival, we investigated whether RC-induced autophagy promotes cell death. The results show that autophagy inhibitors CQ and 3-MA interrupted RC-induced cell death. These results suggest that RC induces apoptosis and autophagic cell death. Because NF-κB is constitutively activated in many cancers, including both solid and hematopoietic malignancies [32], we next investigated whether RC influences the NF-κB pathway in tumor cell lines. The results demonstrate that RC is a new natural inhibitor using in vitro and in vivo experiments as follows: a) the expression of TNF-α- and LPS-induced NF-κB-associated proteins are decreased by RC; b) RC inhibits the expression of TNF-α- and LPS-induced NF-κB-responsive genes; c) the translocation of the p65 induced by TNF-α is interrupted by RC; d) RC not only inhibits the expression of proinflammatory cytokines but also prolongs the survival time of mice in the mouse endotoxin shock model. Furthermore, we also explored where RC acts in the NF-κB signaling pathway. NF-κB-dependent luciferase assay showed that RC inhibits NF-κB activation by overexpressing NF-κB-associated key protein (MyD88, TRAF2, TRAF6, TAK1, TAB1, TAB2, IKKβ or p65), which indicates that RC inhibits the activation of NF-κB pathway upstream of the p65 protein. NF-κB is widely regarded as a modulator of tumor promotion for its ability to suppress cell death pathways, decrease the sensitivity of tumor cells to apoptosis, and promote cell growth [33,34]. Thus, we investigated the correlation between the cytotoxicity and NF-κB inhibited activity of RC and found that RC induces cell death involving in NF-κB pathway. Finally, the in vivo anti-tumor activities of RC were evaluated in HCT116 and HepG2 tumor models. We also investigated the relationship between these effects and apoptosis, autophagy and NF-κB in the meantime. All these results demonstrate that RC inhibits tumor growth and induces apoptosis and autophagy with inhibition of NF-κB in vivo.

**Figure 9:**
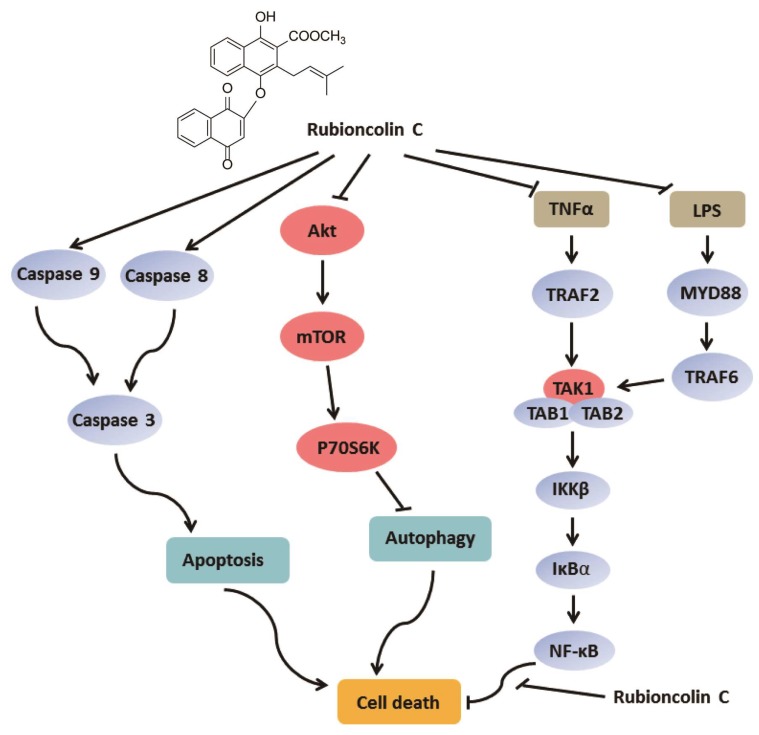
Proposed anti-tumor mechanism of rubioncolin C.

In summary, the present study focused on RC with unique structure and preferable cytotoxicity, a naphthohydroquinone dimer isolated by us from *Rubia podantha*. We investigated its underlying anti-tumor mechanism for the first time and found that RC induced apoptotic and autophagic cell death and inhibited the Akt/mTOR/P70S6K signaling pathway in cancer cells. Furthermore, we also observed that RC inhibited the NF-κB activation upstream of the p65 protein, which promoted RC-induced cell death. In addition, RC prevented LPS-induced endotoxin shock, and suppressed tumor growth through inducing apoptosis and autophagy and inactivating NF-κB in vivo. Taken together, these findings firstly clarified the anti-tumor mechanism of RC (Figure 9) and might contribute to the future development of RC as a new therapeutic agent for treating cancer.

## Supplementary Materials

The following are available online at https://www.mdpi.com/2073-4409/8/12/1593/s1, Figure S1: 17 naphthohydroquinone dimers isolated from *Rubia* plants; Figure S2: The HPLC analysis of rubioncolin C; Figure S3: ^1^H NMR (600 MHz) spectrum of rubioncolin C in CDCl_3_; Figure S4: ^13^C NMR (150 MHz) spectrum of rubioncolin C in CDCl3; Figure S5: ESIMS spectrum of rubioncolin C; Figure S6: Statistical analysis of quantification for Western Blotting data. Table S1: Primers used for quantitative RT-PCR assays.
