# Supplementary material for: Natural Naphthohydroquinone Dimer Rubioncolin C Exerts Anti-Tumor Activity by Inducing Apoptotic and Autophagic Cell Death and Inhibiting the NF-κB and Akt/mTOR/P70S6K Pathway in Human Cancer Cells
Source: Cells. 2019 Dec 7;8(12):1593. doi: 10.3390/cells8121593 (PMC6953124; doi:10.3390/cells8121593)
Supplement: Supplementary file 1 [file cells-08-01593-s001.pdf]

Supplementary material

Natural Naphthohydroquinone Dimer Rubioncolin C Exerts Anti-tumor Activity by Inducing Apoptotic and Autophagic Cell Death and Inhibiting NF-κB and Akt/mTOR/P70S6K Pathway in Human Cancer Cells

Jia Wang [1], Ling Li [1], Jing Wang, Lihua Song, Ninghua Tan * and Zhe Wang *,

Department of TCMs Pharmaceuticals, School of Traditional Chinese Pharmacy, China Pharmaceutical University, Nanjing 211198, People's Republic of China

[1] These authors contributed equally to this work

Corresponding Authors

*Tel.: +86 25 86185772. E-mail address: nhtan@cpu.edu.cn (N.H. Tan), wangzhe@cpu.edu.cn (Z. Wang)

Figrue S1. 17 naphthohydroquinone dimers isolated from *Rubia* plants

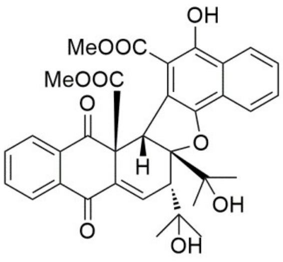

rubioncolin A

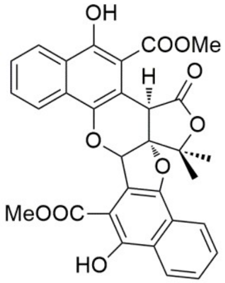

rubioncolin B

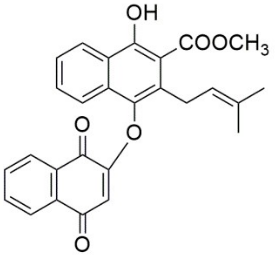

rubioncolin C

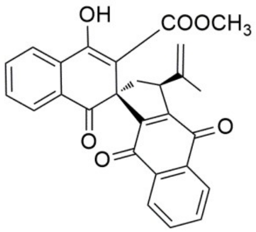

rubioncolin D

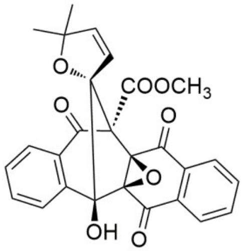

rubialatin A

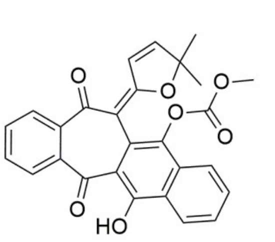

rubialatin B

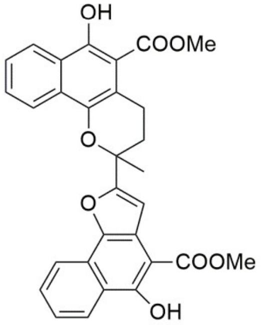

naphthhydroquinone dimer A

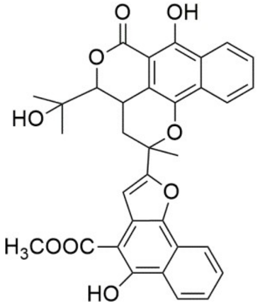

naphthhydroquinone dimer B

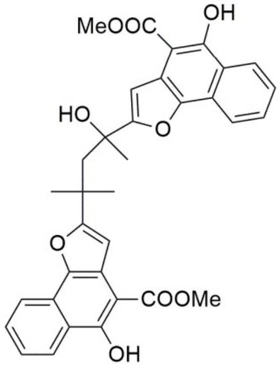

naphthhydroquinone dimer C

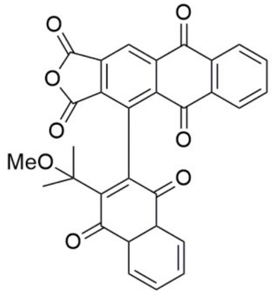

naphthhydroquinone dimer D

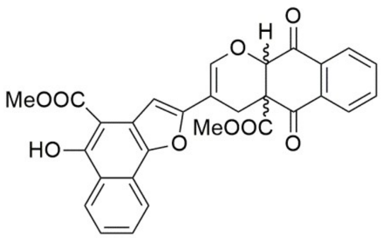

naphthhydroquinone dimer E

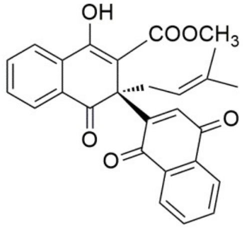

rubipodanone A

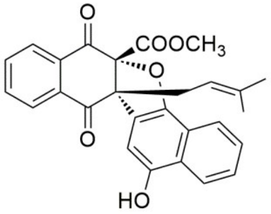

rubipodanone B

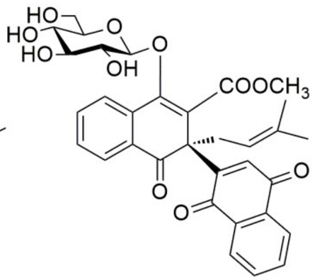

rubipodanone C

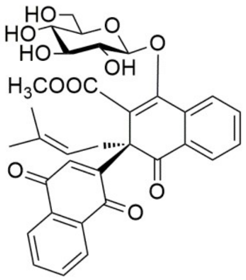

rubipodanone D

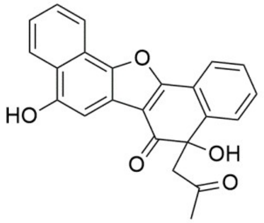

rubiaquinone A

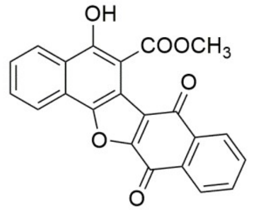

Methyl-5-hydroxy-dinaphtho [1,2-2',3']furan-7,12-dione-6-carboxylate

Figrue S2. The HPLC analysis of rubioncolin C

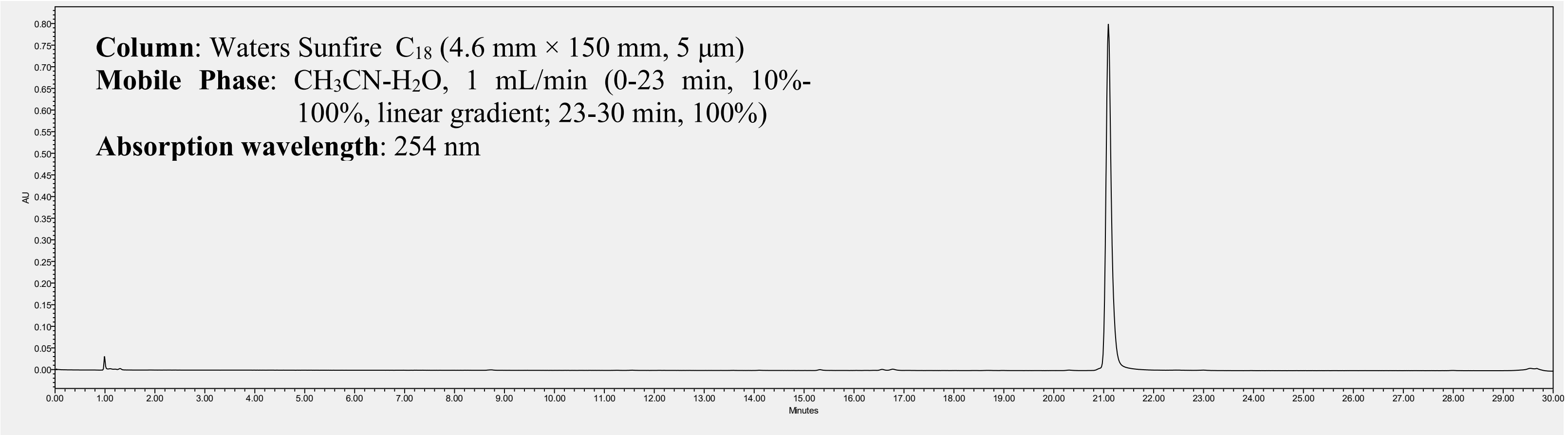

Figure S3. ^{1}H NMR (600 MHz) spectrum of rubioncolin C in $CDCl_3$.

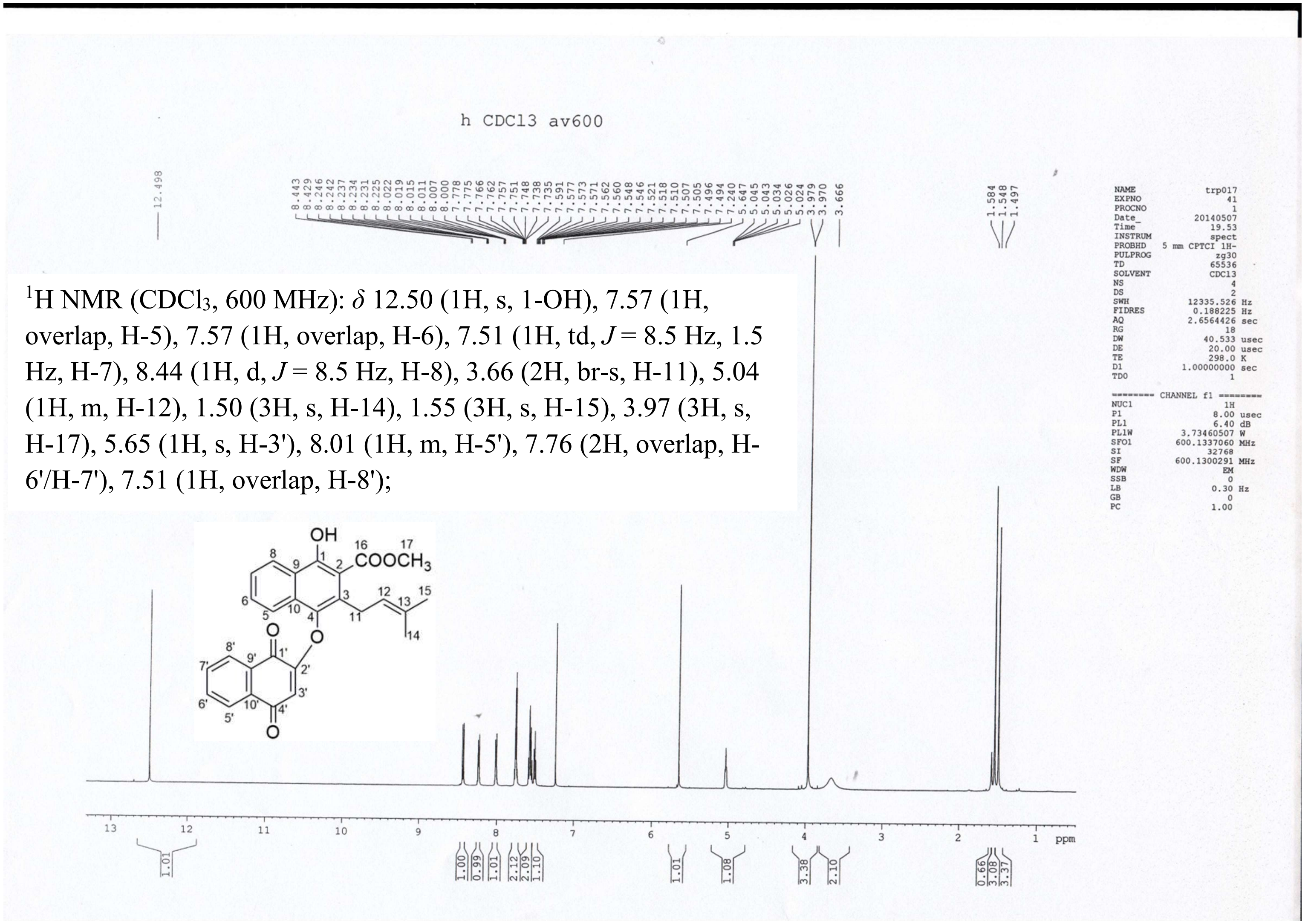

Figure S4. ^{13}C NMR (150 MHz) spectrum of rubioncolin C in $CDCl_3$.

^{13}C NMR ($CDCl_3$, 150 MHz): δ 159.9 (C-1), 105.9 (C-2), 128.9 (C-3), 138.5 (C-4), 121.2 (C-5), 131.0 (C-6), 125.0 (C-7), 122.6 (C-8), 125.0 (C-9), 129.5 (C-10), 27.8 (C-11), 126.5 (C-12), 132.7 (C-13), 18.3 (C-14), 25.7 (C-15), 172.4 (C-16), 52.9 (C-17), 180.0 (C-1'), 161.0 (C-2'), 114.1 (C-3'), 185.0 (C-4'), 126.5 (C-5'), 134.7 (C-6'), 133.8 (C-7'), 127.0 (C-8'), 131.4(C-9'), 132.2 (C-10');

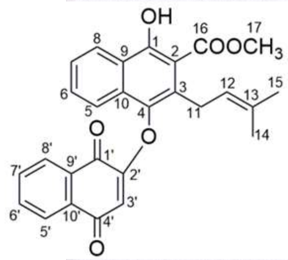

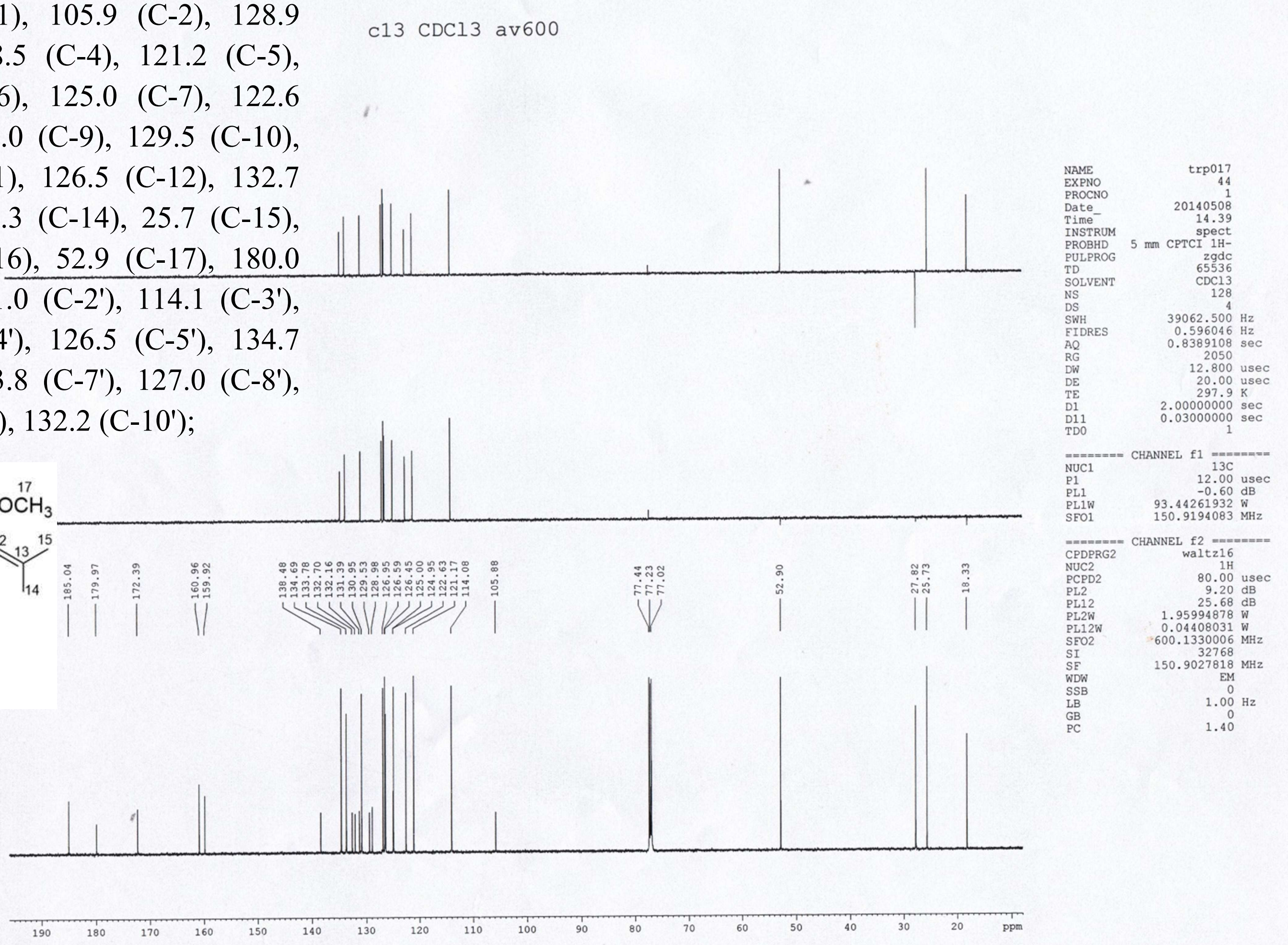

Figure S5. ESIMS spectrum of rubioncolin C

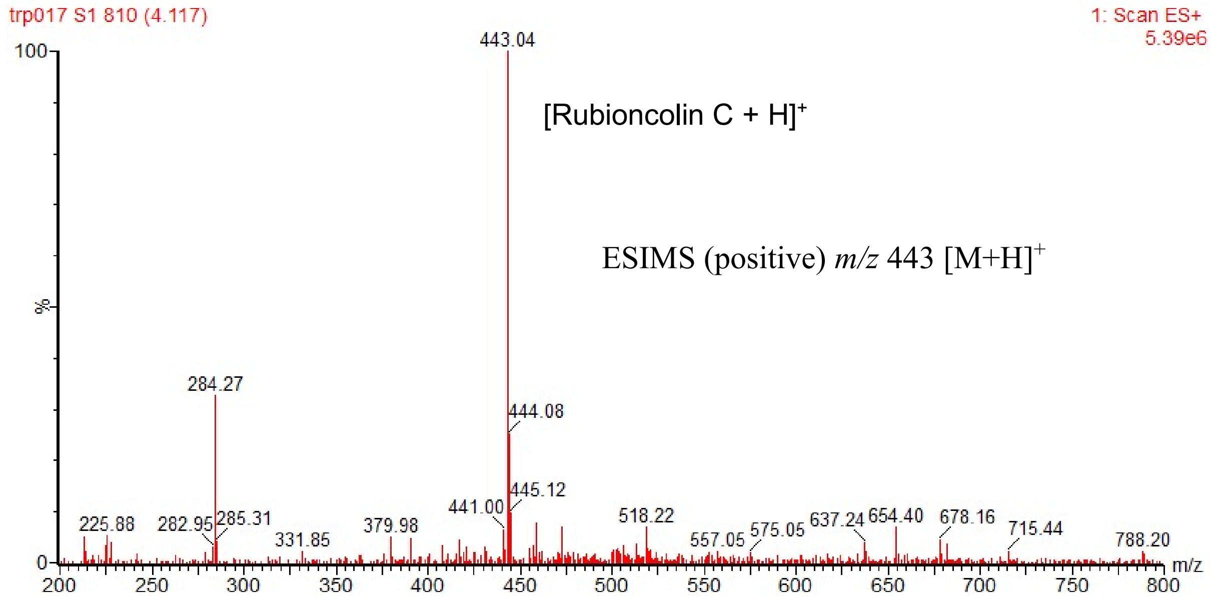

Figure S6: Statistical analysis of quantification for figure 1D (A), figure 2A (B), figure 2B (C), figure 3A (D), figure 3B (E), figure 3G (F), figure 3H (G), figure 4B (H), figure 4C(I) and figure 4H (J). *, $p < 0.05$; **, $p < 0.01$, ***, $p < 0.001$

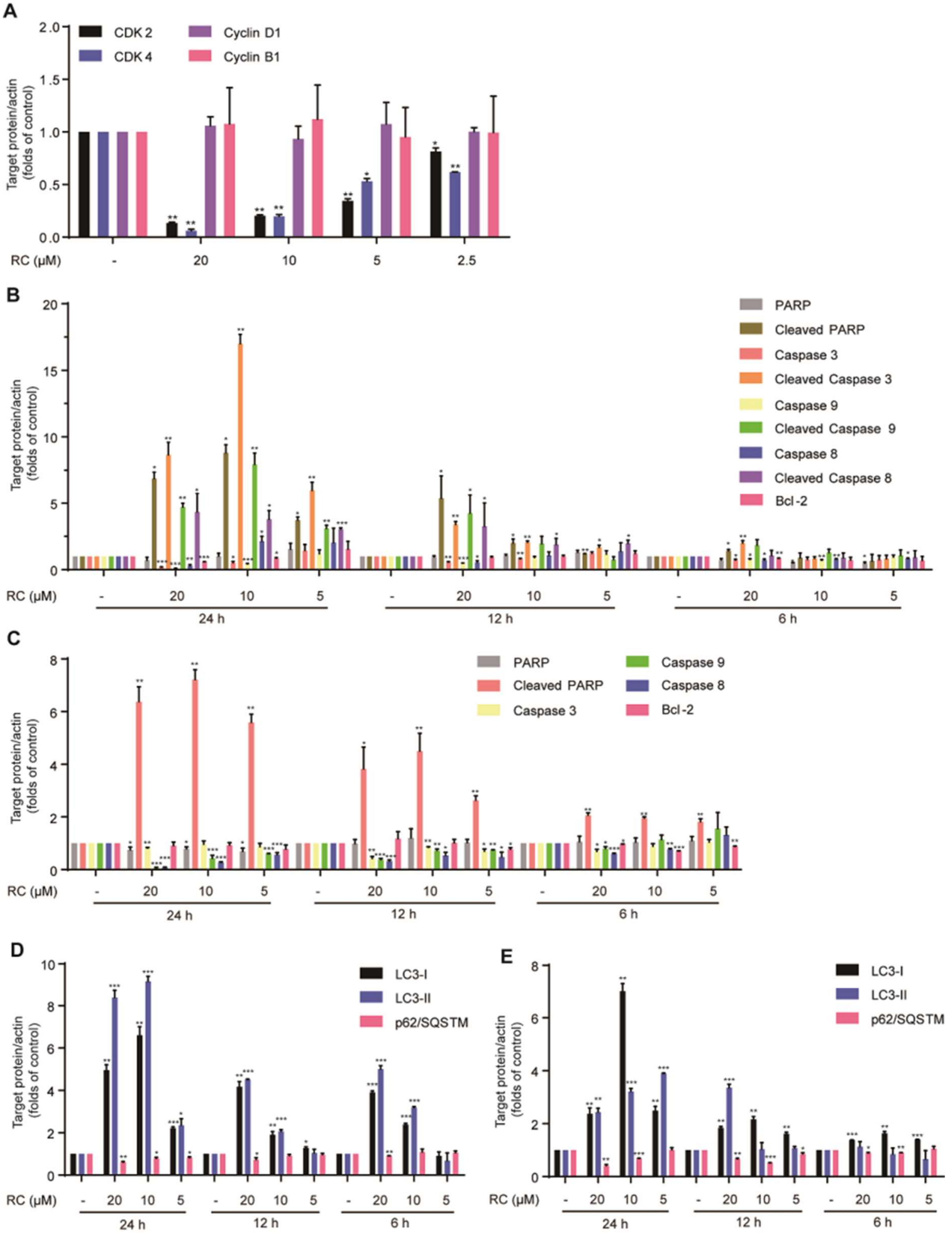

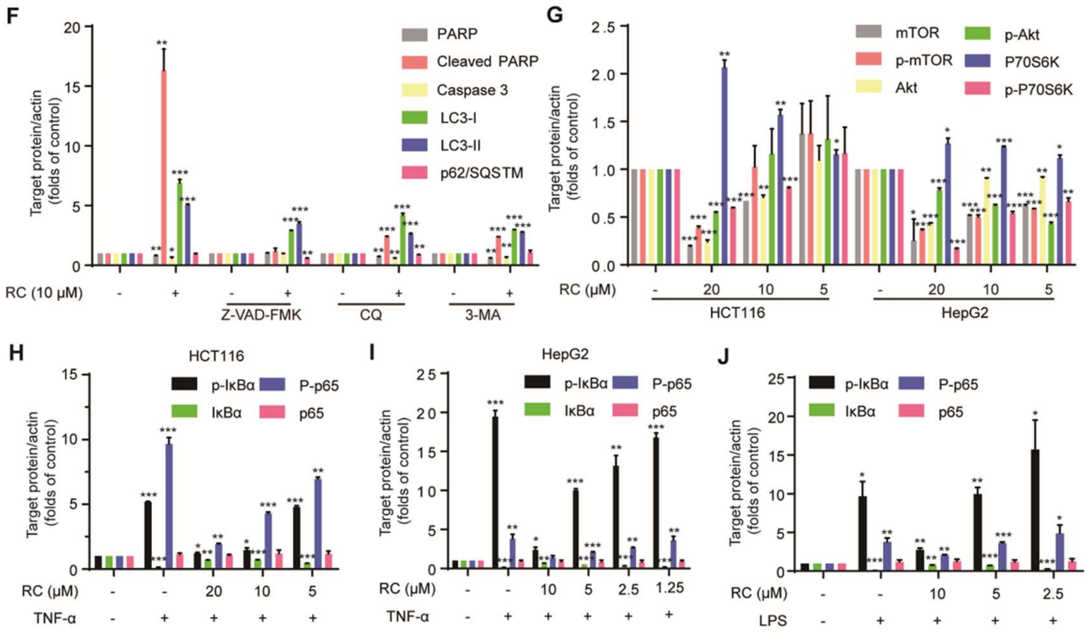

Table S1. Primers used for quantitative RT-PCR assays

Genes	Forward primer (5' - 3')	Reverse primer (5' - 3')
IL-8 **(Homo sapiens)**	GGCAGCCTTCCTGATTTCTG	CTTGGCAAAACTGCACCTTCA
MCP-1 **(Homo sapiens)**	TCGCCTCCAGCATGAAAGTC	GGCATTGATTGCATCTGGC
A20 **(Homo sapiens)**	GCGTTCAGGACACAGACTTG	GCAAAGCCCCGTTTCAACAA
GADPH **(Homo sapiens)**	CGGAGTCAACGGATTTGGTC	GACAAGCTTCCCGTTCTCAG
IL-6 (mus musculus)	GAGAGGAGACTTCACAGAGGATAC	GTACTCCAGAAGACCAGAGG
TNF-α (mus musculus)	CATCTTCTCAAAATTCGAGTGACAA	CCAGCTGCTCCTCCACTTG
GADPH (mus musculus)	GAAGGGCTCATGACCACAGT	GGATGCAGGGATGATGTTCT
